# Steamships and Whiskey

**Published:** 1874-05

**Authors:** 


### STEAMSHIPS AND WHISKEY.

As many vessels have been lost on account of drunken officers, it ought to be known to the traveling public that the Cunard Company have made an imperative rule that if any officer is known to drink liquor during a voyage, he shall be dismissed the service. All the officers are kept under a supervision of which they have no knowledge. The enforcement of the rule that no officer should use liquor while on a voyage has led to the dismissal of one of the most competent captains of the line. If the traveling public will patronize those lines of steamers only which follow the admirable lead of the veteran "Cunarders," a most important step will have been taken in the direction of temperance. Perhaps, after all, it is in indirect ways like these that intemperance may be lessened. If it were generally understood that no man would be permitted to act as an officer on any line of steamers who did not practice total abstinence, a very great change would soon take place in the social habits of sea-faring men. A Boston fire insurance company has lately introduced a rule that they will insure no building in which liquor is retailed, nor the building immediately adjoining.

Suppose railway corporations were to employ no man who used liquor as a beverage, and banks and merchants would employ only temperance clerks, a step would be taken in the direction of temperance more effectual than any form of pledge.

---

A New Hampshire editor, while recently traveling, had his wallet abstracted from his pocket by an adroit pickpocket while indulging in a short nap. The thief was so disgusted with the result of his exploit, that he returned the plunder by express, to the address written inside the wallet, with the following note:

"You miserabil skunk, hear's your pockit book. I don't keep no such. For a man dressed as well as you was to go round with a wallit with nothin' in it but a lot of noospapur stamps an' a pass from a ralerode directur, is a contempterble impersition on the public. As I hear your a editur, I return your trash. I never robs any ony gentlemen."

